# Scalp-Implanted Ultra-Wideband Circularly Polarized MIMO Antenna for Biotelemetry Systems

**DOI:** 10.3390/s24237522

**Published:** 2024-11-25

**Authors:** Zhiwei Song, Youwei Shi, Xianren Zheng, Yuchao Wang

**Affiliations:** The School of Electrical Engineering, Xinjiang University, Shengli Road 666#, Urumqi 830046, China; shiyower@163.com (Y.S.); 107552304503@stu.xju.edu.cn (X.Z.); 107552204406@stu.xju.edu.cn (Y.W.)

**Keywords:** biotelemetry, circular polarization, multiple-input multiple-output, polarization diversity, ultra wideband

## Abstract

This paper presents an innovative, compact, dual-element, implantable, ultra-wideband, circularly polarized multiple-input multiple-output (MIMO) antenna designed to operate within the 2.45 GHz industrial, scientific, and medical band, and both of its radiating units are circularly polarized antennas with polarization diversity. Specifically, antenna-1 exhibits left-handed circular polarization properties, while antenna-2 demonstrates right-handed circular polarization properties. The slots in the radiating patch and ground plane help the antenna achieve 690 MHz (2.14–2.83 GHz) ultra-wide bandwidth characteristics and circularly polarized characteristics. Additionally, a slit connecting two U-slots on the ground plane allows the antenna to achieve a wide effective circularly polarized axial ratio bandwidth of 400 MHz (2.23–2.63 GHz). The antenna is compact, with dimensions of 0.065 × 0.057 × 0.0042 λ_0_^3^ (λ_0_ represents the free-space wavelength corresponding to the lowest operating frequency). The proposed antenna system’s performance was evaluated with a seven-layer homogeneous human head model, a real human head model, and minced pork. This evaluation revealed that the antenna attained a peak gain of −24.1 dBi and an isolation level of 27.5 dB. Furthermore, the assessment included the antenna’s link margin (LM), key MIMO channel characteristics, and Specific Absorption Rate (SAR) metrics. The results indicate that the antenna performs exceptionally well.

## 1. Introduction

Recently, the demand for implantable medical devices (IMDs) in the healthcare industry has surged. Significant advancements have been made in applications like capsule endoscopy, blood glucose monitoring, cardiac pacemakers, intra-oral tongue actuation systems, and intracranial pressure monitoring [1,2,3,4,5]. This increase is driven by the capability of IMDs to detect biological signals within the body and convert them into microwave signals for transmission to an external receiver. IMDs typically incorporate a variety of components, such as sensors, circuit boards, power sources, and antennas [6]. Among these elements, the implanted antenna is particularly critical, as it facilitates effective data communication with a receiver that can be positioned several meters from the human body. Nonetheless, the design of implantable antennas involves overcoming multiple challenges. Most of the current IMDs use single-input single-output (SISO) antennas, including dual/multi-band circularly polarized (CP) antennas [7,8] and ultra-wideband (UWB) antennas [9], but these designs have only one antenna for data transmission and reception; thus, their transmission rates are limited. In addition, due to the complexity and frequency dependence of the human body’s organization, the electromagnetic (EM) characteristics greatly affect the antenna’s performance, and the SISO system is relatively weak in terms of anti-interference capability in the face of signal interference. Since path loss increases with higher operating frequencies, this significantly impacts the communication range of SISO antennas, especially for high-data-rate communications. The newly developed MIMO antenna system shows significant promise in overcoming the constraints of SISO antennas and improving the efficiency of spectrum utilization. The application of MIMO antenna technology in biotelemetry systems has the advantage of improving the communication capacity and reliability compared with the traditional SISO system, but it also faces some unique challenges, which need to be solved in the design of the space, power consumption, signal processing, and interference management in order to achieve stable and reliable communication.

When employing MIMO antenna systems, it is possible to enhance channel capacity (CC) without the necessity for extra power or frequency resources. This is achieved by employing spatial multiplexing techniques, which enable the transmission and reception of data in parallel through multiple antennas. Consequently, this method boosts CC without requiring additional power or frequency resources. MIMO antenna technology has a large advantage in improving the wireless communication capacity and transmission rate. Although the traditional MIMO technology is mature, there are still some limitations in some dynamic or complex scenarios. Recently, some scholars have proposed realizing the channel estimation of the movable MIMO antenna system through tensor decomposition [10]. This technology brings new possibilities in the channel estimation of mobile MIMO antenna systems. More recently, millimeter-wave and terahertz bands have also become attractive for ultra-wideband transmission. A channel training-aided target-aware framework was developed in [11] to facilitate efficient resource sharing for THz-massive MIMO communication. In [12], the spatial broadband effect and the associated beam skew problem in MIMO communication were addressed by constructing a massive MIMO channel model. In addition, the MIMO antenna power leakage problem was significantly reduced and inter-user interference was mitigated by an energy-focused window approach in [13].

In [14], a four-element, implantable, linearly polarized (LP) MIMO antenna, designed for the 2.4 GHz industrial, scientific, and medical (ISM) band and utilizing electromagnetic bandgap (EBG) technology, is introduced. This antenna exhibits a fractional bandwidth (FBW) of 18.64% (ranging from 2.14 to 2.58 GHz) and demonstrates a mutual coupling of no more than −15.99 dB within the ISM band. In [15], a compact MIMO implantable LP antenna system is introduced, engineered for operation within the Medical Implantation Communication Service (MICS) band (402–405 MHz) and the ISM band (433.1–438.8 MHz). This design exhibits an FBW of 33.9% (355–500 MHz) and isolation exceeding 26 dB. In [16], a MIMO antenna with two ports was developed for the 403 MHz MICS band, featuring a spiral resonator design. This design achieves an analog FBW of 35.9%, in addition to a maximum isolation of 26 dB in the 433 MHz ISM band. Despite its performance, the antenna’s size is relatively large, at 307 mm^3^. In [17], a quadratic MIMO antenna was introduced, which includes a semicircular bent radiator sharing a common ground. This antenna is tuned to operate at 433 MHz and offers an FBW of 38.26% (355–523 MHz). A compact two-port implantable MIMO antenna with a bent resonator, operating at 2.45 GHz, is reported in [18]. This antenna features a bandwidth of 320 MHz and achieves an isolation of over 28 dB at the design frequency. In [19], a novel conformal MIMO antenna was introduced, marking the debut of eigenmode theory in MIMO antenna design. This antenna exhibited an FBW of 15.1% (ranging from 2.26 to 2.63 GHz) and achieved an impressive isolation level of 27.26 dB.

All of the above MIMO antennas are LP antennas, and in complex multipath propagation environments, LP antennas may suffer from multipath interference, resulting in performance degradation. CP MIMO systems offer significant advantages in terms of multipath interference resistance, stable transmission, polarization mismatch resistance, and dynamic adaptability, and they have wider application prospects in mobile communications and dynamic environment applications, ensuring signal strength and connectivity stability without being significantly affected by changes in device attitude. To address the above challenges, [20] introduced a 3D MIMO ground-radiating cube antenna (CA) fed by a coplanar waveguide (CPW), which was circularly polarized by loading a pair of slots, but it was not suitable for IMDs due to its large size. Ref. [21] describes a MIMO antenna with directional mapping and polarization diversity characteristics for capsule endoscopy applications. It operates in the 915 MHz ISM band with 12.35% FBW and 10.35% 3 dB axial ratio bandwidth (ARBW). Though the above implantable MIMO antennas are circularly polarized, they suffer from the limitations such as excessive size or insufficient bandwidth.

To overcome the aforementioned limitations, this paper presents a new, compact, dual-element, implantable, ultra-wideband, circularly polarized MIMO antenna designed for operation at 2.45 GHz. It enables high-speed data transmission without requiring additional power or frequency resources. The antenna has a bandwidth of 28.16% (2.14–2.83 GHz) and a wide effective 3 dB ARBW of 16.3% (2.23–2.63 GHz). It also has an isolation of greater than 27.5 dB and a peak gain of −24.1 dBi. The primary benefits of this MIMO antenna include (1) its highly compact design; (2) the availability of independent channels, which enhance CC; (3) an ultra-wide bandwidth and wide effective axial ratio (AR); and (4) the ability to support polarization diversity.

## 2. Design Methodology

### 2.1. Antenna Geometry

**Figure 1:**
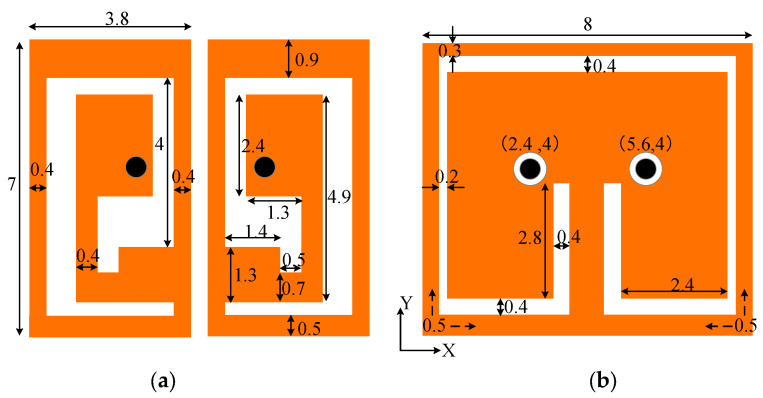

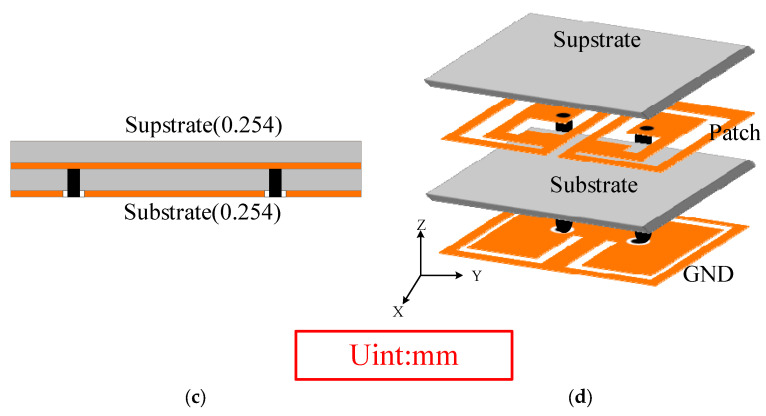
Geometry of the designed dual-unit implantable MIMO antenna: (**a**) Radiating patch. (**b**) Ground plane. (**c**) Side view. (**d**) Decomposed view.

Figure 1a, b depict the configurations and detailed specifications of the radiating and ground planes for the proposed implanted ultra-wideband circularly polarized MIMO antenna. Two radiating patches, each with an equivalent design, are linked to a common ground plane and stimulated by coaxial probes. The probes feature inner and outer diameters measuring 0.6 mm and 0.8 mm, respectively. Figure 1c,d show that the antenna cover layer is selected as Rogers 3010 (*ε_r_* = 10.2, tan*δ* = 0.002) and the dielectric substrate is selected as Rogers 5880 (*ε_r_* = 2.2, tan*δ* = 0.002), and both of them have a thickness of 0.254 mm. The cover layer can effectively avoid mutual interference caused by direct contact between the antenna and other components of the IMD, and at the same time broaden the operating bandwidth of the antenna [22]. The low dielectric constant of the dielectric substrate reduces the propagation speed of the signal in the antenna structure, thus reducing the signal transmission delay, which is important for communication systems requiring high-speed data transmission and low latency. Since the two radiating patches are disconnected, there is no direct current (DC) leakage from one element to the other. However, the fringing field of the antenna can generate capacitive coupling, which varies with the inter-element gap. To ensure minimal coupling, a 0.4 mm gap is maintained between the radiating surfaces.

### 2.2. Simulation Environment and Measurement Setup

**Figure 2:**
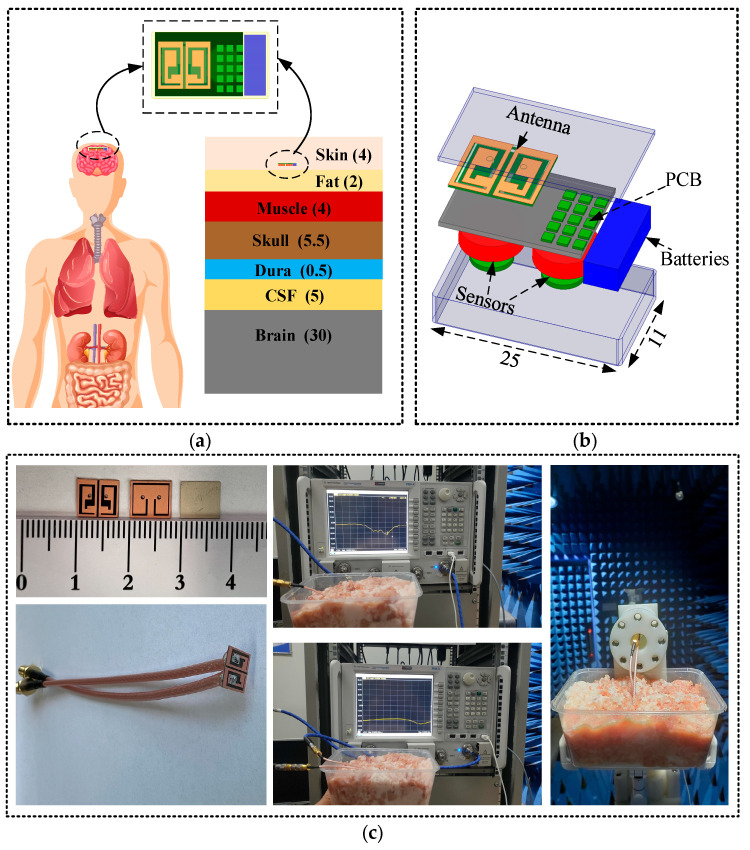
(**a**) Simulation environment, (**b**) IMD architecture, and (**c**) physical and measurement setup for the antenna.

In this research, the proposed MIMO antenna’s simulation and optimized design were conducted utilizing HFSS 2021. As shown in Figure 2a, the simulation was performed in a 7-layer homogeneous human head model and a real human head model in order to be closer to the actual human body, and to analyze the actual working performance of this antenna. The total dimensions of the 7-layer homogeneous human head model are 100 × 100 × 51 mm^3^, and the electrical characteristics of the individual tissues are dependent on the resonant frequency. Table 1 provides details on the specified thickness, along with the relative permittivity (*ε_r_*) and conductivity (*σ*) for each layer of the human head model at 2.45 GHz [23,24]. The device was implanted at a 3 mm depth within both the 7-layer uniform human head model and the actual human head model.

Biomedical implantable antennas designed for use in the human body must be biocompatible [25]. Furthermore, each implantable biomedical device has a designated implantation area and specific architectural requirements [26]. As shown in Figure 2b, an IMD typically includes an antenna, a printed circuit board, sensors, and batteries, all of which are encapsulated in a biocompatible alumina container. Therefore, the biocompatibility of the device is of primary importance. In this work, alumina (*ε_r_* = 9.8) was selected as the biocompatible material for the device housing. The length, width, and thickness of the flatbed device are 25 mm, 11 mm, and 0.2 mm, respectively. It contains a PCB that incorporates surface-mounted components, batteries, sensors, and the new ultra-wideband circularly polarized MIMO antenna. The device enclosures are sealed with epoxy resin to prevent liquid ingress during experimental measurements. The physical antenna and its measurement configuration are shown in Figure 2c. For actual measurements, the entire antenna system was embedded in minced pork to a depth of 3 mm. The S_11_ and S_21_ parameters for the constructed antenna were assessed within minced pork using a vector network analyzer.

### 2.3. Steps in Design Evolution

The key goals in optimizing the antenna design were to (1) expand the FBW, (2) attain high levels of isolation, and (3) achieve CP characteristics with an enhanced ARBW.

The size of the radiating patch can be determined by the following equation:

(1) $$L=\frac{c}{2\times f\sqrt{\varepsilon_{r}}}$$

where the resonant frequency is represented by *f*, *ε_r_* (2.2) is the substrate relative permittivity, the velocity of light is denoted by *c* (3 × 10^8^ m/s), and *L* symbolizes the effective length of the radiation patch.

**Figure 3:**
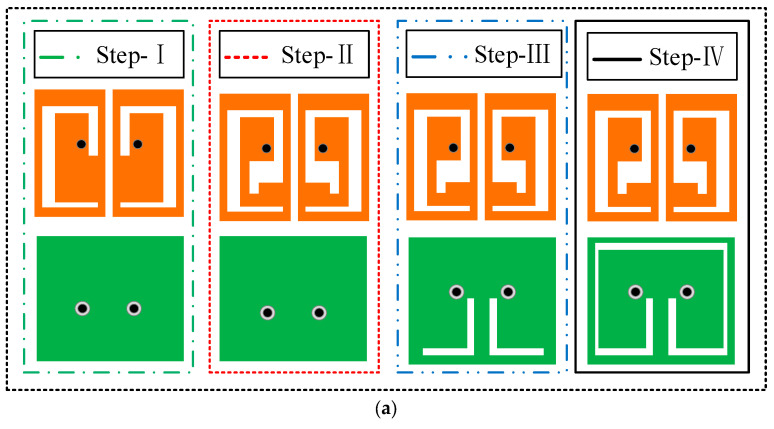

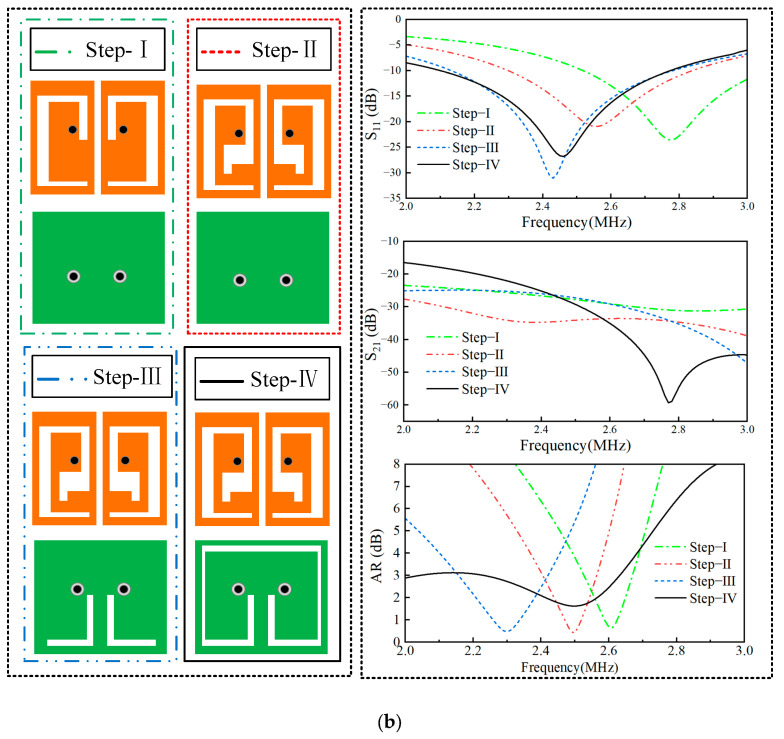
(**a**) Steps in the design evolution process. (**b**) |S_11_|, |S_21_|, and AR simulations of the antenna at each stage of the design evolution.

The MIMO antenna is characterized by a continuous ground plane, which is shared by both radiating units. The design steps are shown in Figure 3a,b, illustrating the comparison of the results of S_11_, S_21_, and AR corresponding to each design step. In Step I, a slot is introduced into the radiating patch, resulting in the antenna resonating at 2.8 GHz. It has isolation greater than 22 dB and a 3 dB ARBW of 0.14 GHz (2.53–2.67 GHz). In Step II, to shift the resonance point and the axial ratio point to the left, two L-shaped rectangular slots are integrated into the ground plane. This modification influences the antenna’s impedance characteristics, resulting in a leftward shift of the resonance point and an expanded bandwidth. In Step III, vertical slits are added at the ends of the L-shaped rectangular slots. These slits introduce additional capacitance to the antenna, increasing its reactance. Consequently, the resonance frequency is lowered, resulting in enhanced impedance matching. However, this modification reduces the antenna’s isolation and the effectiveness of the circularly polarized AR. Thus, in Step IV, a horizontal slot is incorporated to connect the two U-slots in the ground plane. Ground-plane slotting introduces an additional reactance effect on the ground plane, which helps to achieve more stable circular polarization characteristics while increasing the ARBW. The findings reveal that the antenna achieves a resonant frequency of 2.45 GHz and an isolation level exceeding 27.5 dB. Additionally, it achieves an FBW of 28.16% and an effective ARBW of 16.3%.

## 3. Results and Discussion

### 3.1. Comparative Analysis of Main Parameters

**Figure 4:**
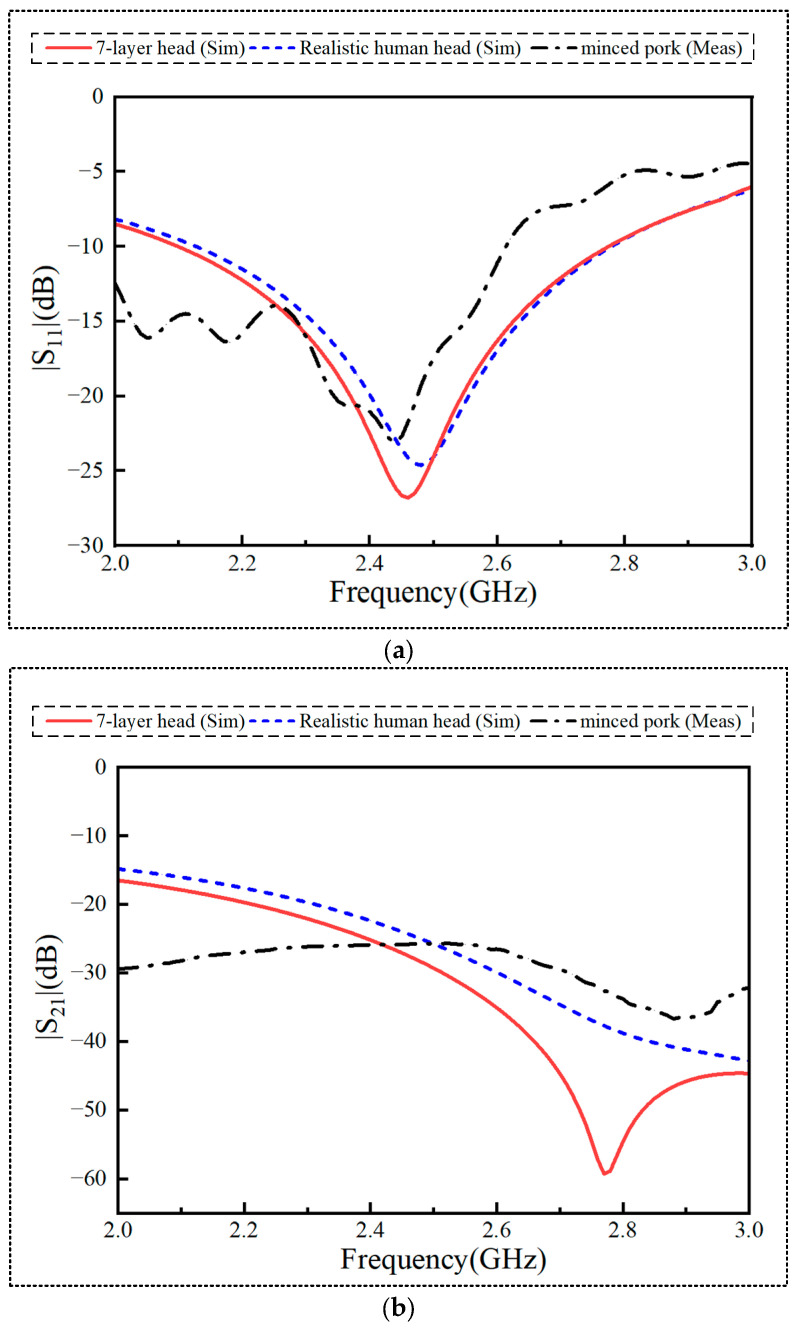

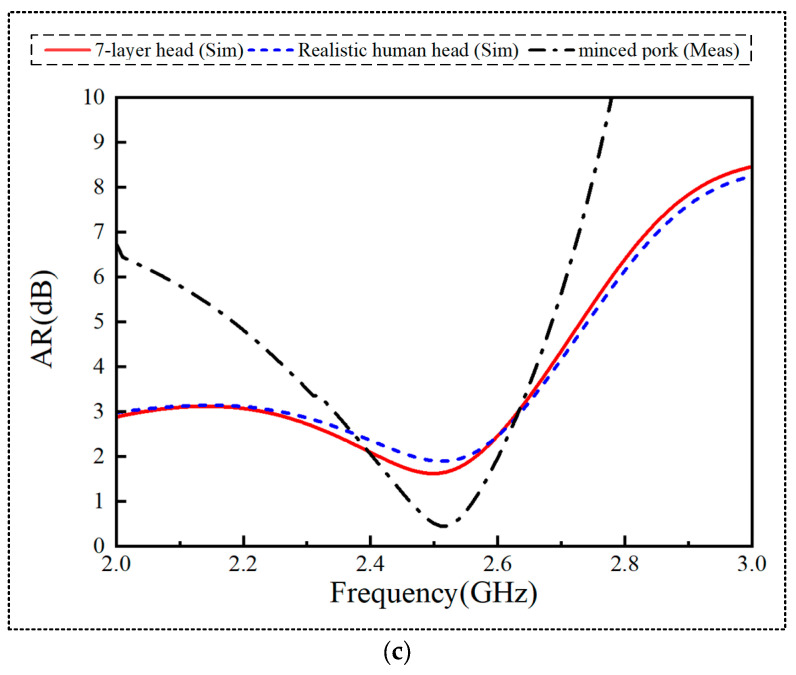
Simulation and test results of the antenna in different environments (**a**) |S11|, (**b**) |S21|, and (**c**) AR.

The proposed antenna’s performance was first examined through simulations and analyses conducted with Ansys HFSS 2021, utilizing both a seven-layer homogeneous human head model and an actual human head model. To corroborate the simulation findings, the fabricated antenna underwent testing in minced pork. Figure 4a–c present the |S_11_|, |S_21_|, and AR parameter results derived from simulations with the seven-layer homogeneous human head model and the real human head model, as well as from the measurements conducted in minced pork. Figure 4a clearly shows that the antenna operates at a resonant frequency of 2.45 GHz, which is within the ISM band (2.4–2.48 GHz). Specifically, in the seven-layer homogeneous human head model, the real human head model, and minced pork, the antenna exhibited resonant frequencies of 2.45 GHz, 2.5 GHz, and 2.4 GHz, respectively. These correspond to FBWs of 28.16% (2.14–2.83 GHz), 26.12% (2.13–2.77 GHz), and 28.16% (1.92–2.61 GHz).

Figure 4b illustrates that, at 2.45 GHz, the |S_21_| value from simulations within the seven-layer homogeneous human head model was −27.5 dB. Conversely, the measured |S_21_| values were consistently below −26.5 dB across the operational frequency range. The minor discrepancy between the simulated and measured results may be due to factors such as the effect of soldering coaxial cables, which can influence the antenna’s surface wave propagation and the reflected field. This could cause surface waves to propagate from one port to the other, thereby affecting the antenna’s performance. It should be noted that, since the two antenna radiating units have similar geometries—|S_11_| = |S_22_| and |S_21_| = |S_12_|—only the results for |S_11_| and |S_21_| are presented in the figure, while |S_22_| and |S_12_| are ignored.

The simulated and measured ARs are illustrated in Figure 4c, where the 3 dB ARBW values obtained from simulation in the seven-layer human head model and the real human head model are close to each other, at 16.3% (2.23–2.63 GHz) and 15.9% (2.25–2.64 GHz), respectively. Additionally, the measured 3 dB ARBW in minced pork reaches 12.2% (2.34–2.64 GHz). It can be noted from the figure that the ARBW of the proposed antenna completely covers the desired ISM band.

### 3.2. Circular Polarization Analysis

(1) *The 3 dB AR*

**Figure 5:**
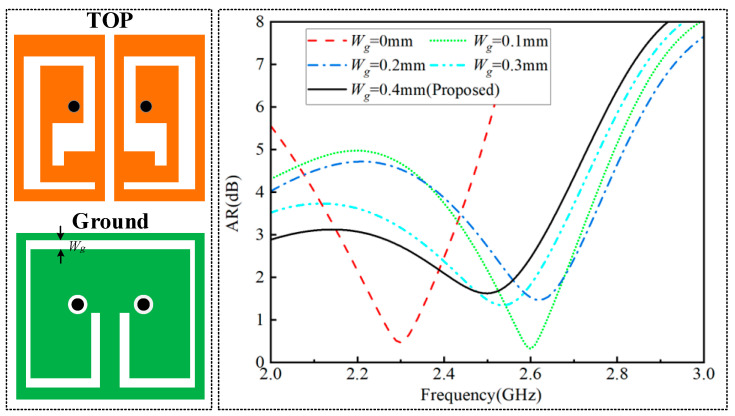
Effect of ground-plane gap width (Wg) on antenna ARBW.

A parametric analysis was performed to assess the impact of the gap width (Wg) in the ground plane on the 3 dB ARBW of the CP antenna, as illustrated in Figure 5. In this study, a slit in the ground plane linked the left and right U-slots, leading to an increase in the 3 dB ARBW. The effective ARBW was further improved by adjusting the width of the slit. By analyzing the proposed antenna ground-plane slit, we selected a slot width of 0.4 mm, as this provides a wider effective ARBW.

(2) *Current Distribution*

**Figure 6:**
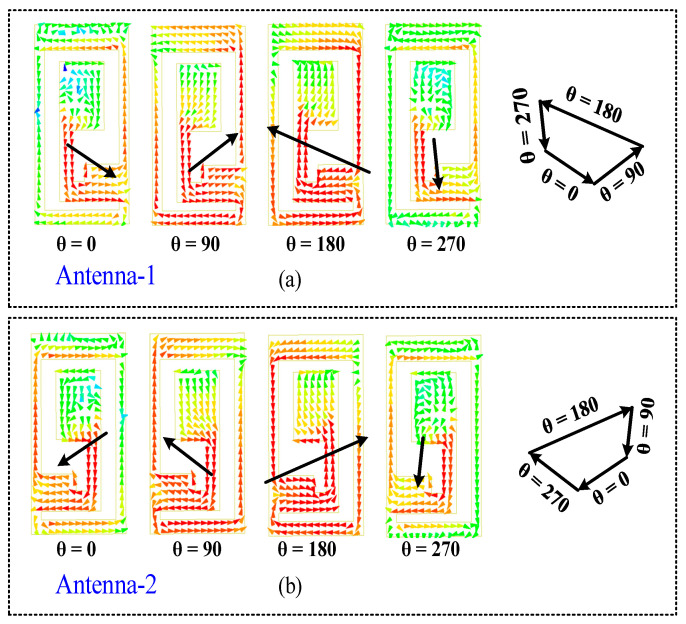
Surface current distribution over the patch of (**a**) antenna-1 at 2450 MHz and (**b**) antenna-2 at 2450 MHz.

Insights into the antenna’s CP characteristics are provided by the distribution of surface currents [27]. Figure 6a shows the current distribution when antenna-1 is active and antenna-2 is passive. It is evident from the figure that the currents in the left-hand radiating patch rotate counterclockwise over time, which corresponds to RHCP. In contrast, Figure 6b illustrates the current distribution when antenna-2 is operational and antenna-1 is inactive. In this scenario, the currents flowing through the right-hand radiating patch revolve in a clockwise direction, indicative of LHCP. In conclusion, the designed MIMO antenna facilitates dual polarization modes and attains polarization diversity at 2.45 GHz. Specifically, antenna-1 is RHCP, while antenna-2 is LHCP.

(3) *Radiation Pattern*

**Figure 7:**
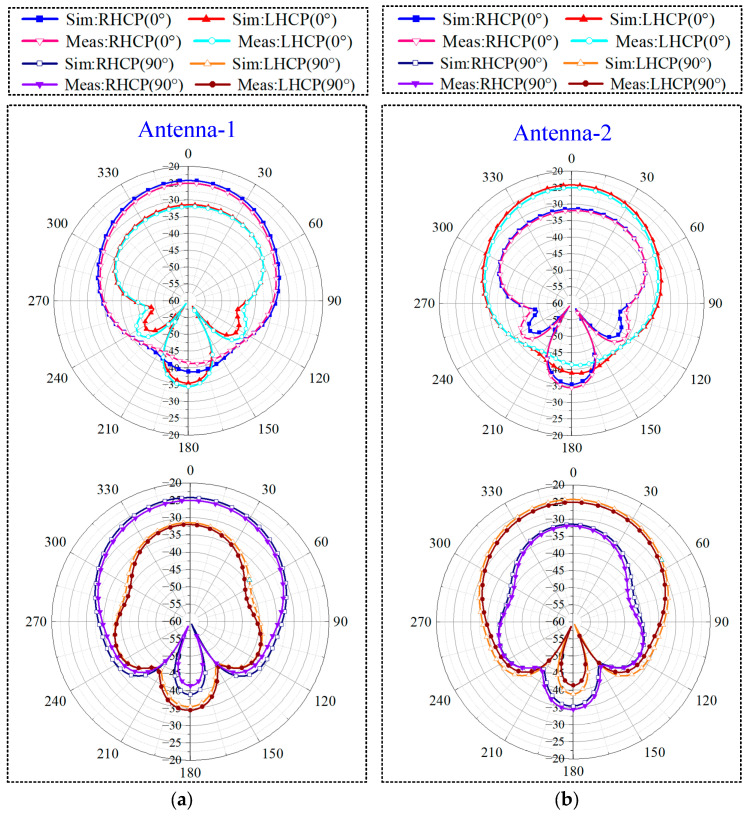
Two-dimensional radiation direction diagrams of (**a**) antenna-1 and (**b**) antenna-2.

Figure 7a,b depict the simulation and measurement outcomes for the CP radiation patterns within the planes defined by φ = 0° and 90°. These outcomes were documented during testing phases conducted within an anechoic chamber, utilizing the dual-antenna configuration. For the measurements, the implantable device was placed in a minced-pork-filled model. One port of the MIMO antenna system was connected to a 50 Ω load resistor, whereas the other port was linked to a spectrum analyzer. A transmitter horn antenna, positioned 3 m from the test antenna, was rotated in 1° steps to collect the radiated power data.

Figure 7a shows the radiation patterns for RHCP and LHCP of antenna-1 at 2.45 GHz, showing that antenna-1 exhibits LHCP. Similarly, Figure 7b shows that antenna-2 exhibits RHCP. Both figures reveal a cross-polarization discrimination (XPD) greater than 8 dB. Thus, the designed antenna achieves polarization diversity at 2.45 GHz by supporting dual polarization modes. Furthermore, the main radiation direction for both antennas is oriented away from the body. The simulated and measured peak gains of the antennas are −24.1 dBi and −25.3 dBi, respectively.

### 3.3. Link Budget for Wireless Communications

The scalp-implantable device aims to transmit collected data to an external receiver, making an *LM* analysis essential to assess the communication capability, transmission distance, and speed of data transmission. The *LM* is usually calculated using the following equation [28]:

(2) $$LM=Link\frac{c}{N_{0}}-Required\frac{c}{N_{0}}$$

(3) $$Link\frac{c}{N_{0}}=P_{t}+G_{t}-L_{f}-L_{p}+G_{r}-N_{0}$$

(4) $$Required\frac{c}{N_{0}}=\frac{E_{b}}{N_{0}}+10\mathrm{lg}B_{r}-G_{c}+G_{d}$$

(5) $$L_{f}=20\log_{10}(\frac{4\pi d}{\lambda })$$

(6) $$N_{0}=10\log_{10}(k)+10\log_{10}(T_{i})$$

(7) $$T_{i}=T_{0}(NF-1)$$

**Figure 8:**
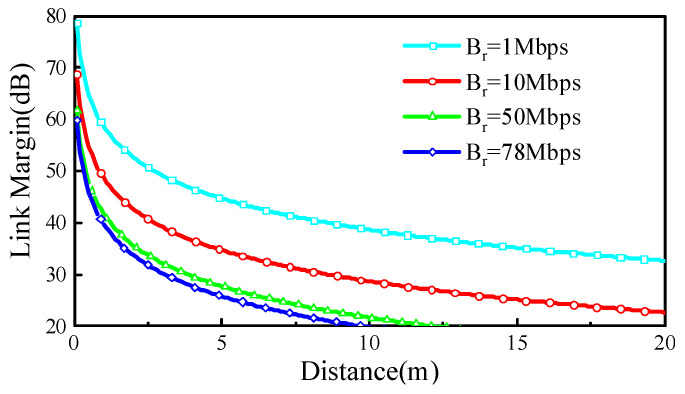
Link margin for different transmission rates at 2450 MHz.

All of the variables and values utilized in the calculations are detailed in Table 2. In the link communication setup, the implanted CP MIMO antenna functions as the transmitting antenna, while an external CP dipole antenna with a gain of 2.15 dBi serves as the receiving antenna. Given that both antennas exhibit CP properties, the polarization mismatch loss is assumed to be negligible at 0 dB. Effective communication between the two antennas is ensured when the *LM* exceeds 0 dB. In this work, a minimum reference margin of 20 dB was used to guarantee stable and seamless communication. Figure 8 shows that, at an operating frequency of 2.45 GHz, the *LM* decreases as the communication distance increases. The tested data transmission rates were 1, 10, 50, and 78 Mbps, with the maximum transmission distance reaching 9.6 m at a bit rate of 78 Mbps. Therefore, the proposed ultra-wideband CP MIMO antenna is highly suitable for use in scalp-implantable devices.

### 3.4. Channel Parameters

(1) Envelope Correlation Coefficient (*ECC*): The *ECC* is a crucial metric that indicates the diversity and coupling performance of a multiple-antenna configuration in a MIMO system. It is used to verify the system’s independence regarding separation performance. In the ideal case, the value of *ECC* is 0, and the channel of MIMO does not want to be turned off at all, but in practical applications, *ECC* < 0.5 is usually considered acceptable. *ECC* can be calculated using either S-parameters or far-field radiated directional maps, and the S-parameter method of Equation (8) was used for *ECC* calculations in this work [29]:

(8) $$ECC=\frac{|\iint_{4\pi }({\vec{An}}_{i}(\theta ,\phi ))\times ({\vec{An}}_{j}(\theta ,\phi ))d\Omega {|}^{2}}{\iint_{4\pi }|({\vec{An}}_{i}(\theta ,\phi )){|}^{2}d\Omega \iint_{4\pi }|({\vec{An}}_{j}(\theta ,\phi )){|}^{2}d\Omega }$$

where ${\vec{An}}_{i}(\theta ,\phi )$ is the 3D radiation pattern of antenna-1 and ${\vec{An}}_{j}(\theta ,\phi )$ is the 3D radiation pattern of antenna-2; Ω is the solid angle.

**Figure 9:**
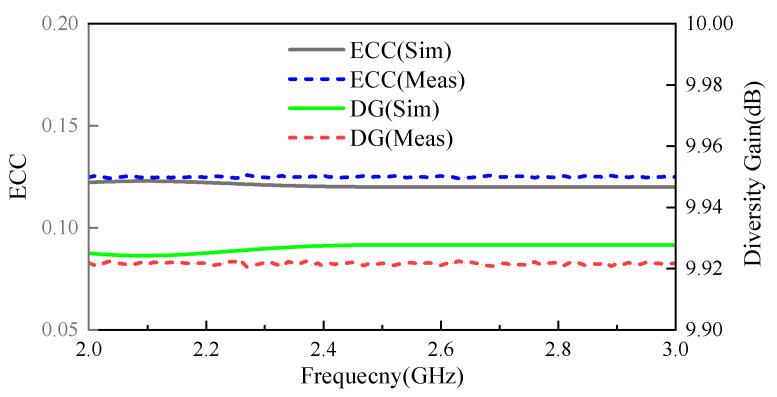
MIMO channel parameters: ECC and DG.

Figure 9 illustrates the plot of the *ECC* value against frequency. It should be noted that the antenna system’s overall *ECC* remains below 0.03, with a specific measurement of 0.02 at the 2.45 GHz frequency point. The sufficiently low *ECC* value confirms the excellent diversity performance, making the antenna system suitable for implantable devices with high data rates, such as scalp IMDs.

(2) Diversity Gain (*DG*): Moreover, the *DG* reflects how the diversity scheme influences the transmission power. A *DG* value of 10 dB is considered optimal, indicating a perfectly uncorrelated channel. The *DG* for a MIMO antenna can be calculated as follows [30]:

(9) $$DG=10\sqrt{1-(ECC{)}^{2}}$$

From the above equation, it is evident that the *ECC* value determines the *DG* value. Specifically, as the *ECC* increases, the *DG* decreases, and vice versa.

Figure 9 shows the *ECC* and *DG* of the antenna across various frequencies. The results show that the simulated and measured *ECC* of this antenna system is less than 0.03 over the entire range of the target operating band, that the measured *DG* is greater than 9.995 dB over the entire operating band, and that this MIMO antenna system is more independent.

(3) Channel Capacity (*CC*): The primary goal of MIMO antenna systems is to enhance capacity without requiring extra spectral or power resources. The bit error rate (BER) of the antenna system increases as the number of both transmitting and receiving antennas rises, provided that the channels are uncorrelated. However, in practical applications, achieving completely uncorrelated channels is not feasible. The *CC* is affected by both the number of antennas and the degree of channel correlation. Achieving high *CC* requires an increased number of antennas and high isolation. The *CC* for an *N* × *N* MIMO antenna system can be described by the following formula [31]:

(10) $$CC=\mathrm{lo}g_{2}(\det[I_{N}+\frac{SNR}{N}HH^{*}])$$

where $I_{N}$ represents the identity matrix, *H* includes the amplitude-phase information of the antenna in the form of a channel matrix, and H* is the covariance matrix of *H*.

**Figure 10:**
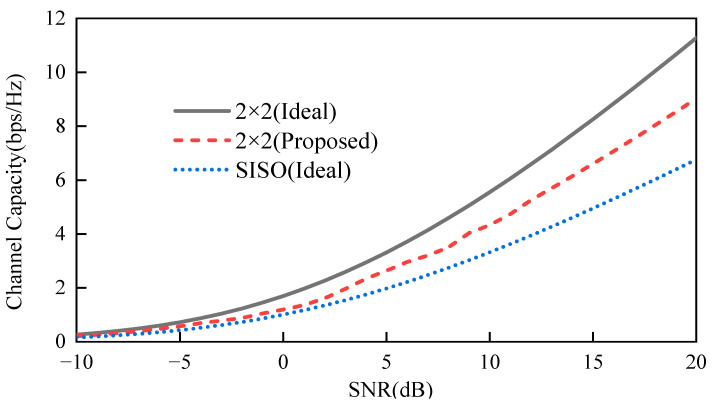
Comparison of CC with SNR change.

In this study, the channel matrix was derived from the radiation direction map of the antenna [32]. Figure 10 shows the *CC* in bps/Hz plotted against the signal-to-noise ratio (SNR) in decibels for ideal SISO, ideal 2 × 2, and the proposed 2 × 2 antennas. When the SNR hits 20 dB, the capacity of an ideal MIMO antenna system significantly outperforms that of an ideal SISO system. As can be seen from the figure, the channel capacity of the designed MIMO antenna system exceeds that of the ideal SISO antenna in the overall range. As a result, this antenna is exceptionally well suited for high-data-rate biomedical IMD applications.

### 3.5. Specific Absorption Rate of Energy Deposition

**Figure 11:**
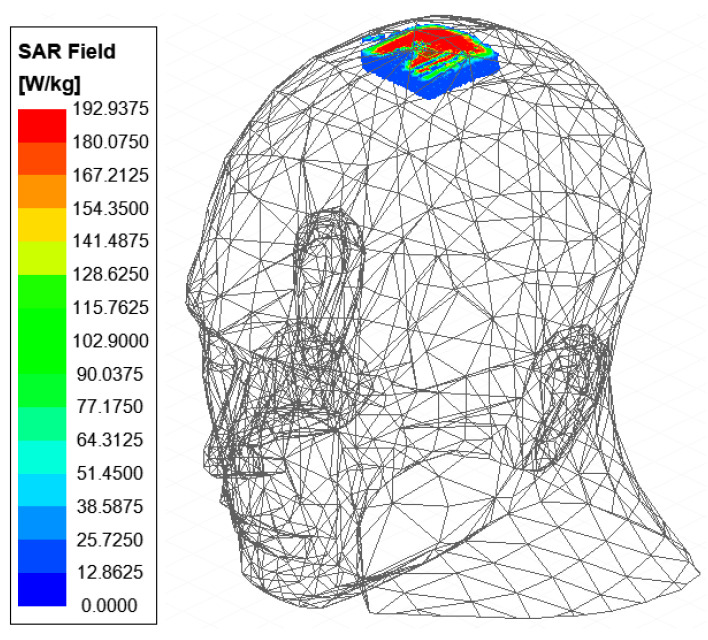
SAR over 10 g of tissue at 2450 MHz when both ports are powered with 1 W in a real human head.

Implantable devices transmit data to external devices via electromagnetic waves, and this propagation can be absorbed by human tissues, thereby causing damage. SAR signifies the quantity of electromagnetic energy absorbed by living beings, serving as a critical metric for evaluating the health implications associated with IMDs. According to the IEEE standard, for an input power of 1 W, the 1 g averaged SAR must not go beyond 1.6 W/kg, and the 10 g averaged SAR should remain below 2 W/kg [33]. To assess the antenna’s SAR, the IMD was implanted at a depth of 3 mm within a real human head model, with both feeding ports of the MIMO antenna simultaneously excited with an input power of 1 W. As shown in Figure 11, the designed antenna exhibited a maximum SAR of 192.9 W/kg in each 10 g of tissue, with each individual antenna achieving a maximum SAR of 96.45 W/kg in each 10 g of tissue. This assessment considers the implantation depth and the overall impact on the surrounding 10 g of tissue. According to IEEE standards, the suggested antennas are capable of managing an input power level of up to 16.59 mW safely. In practice, the input power for IMDs is restricted to 16 dBm to prevent interference with other devices [34]. Therefore, the suggested antenna can operate safely with an input power of up to 16.59 mW (12.2 dBm).

Table 3 presents a summary of the proposed antenna’s performance alongside a comparison with recent studies on implantable MIMO antennas. The table clearly indicates that the design and investigation of the proposed MIMO antenna utilized a seven-layer head model. In addition, the antenna has a wider FBW and wider effective ARBW under the conditions of antenna miniaturization and polarization diversity.

## 4. Conclusions

This paper introduces a miniaturized two-unit implantable ultra-wideband CP MIMO antenna with two individually excited radiating patches: antenna-1 operates with LHCP and antenna-2 with RHCP, achieving polarization diversity at 2.45 GHz. The radiating patches are separated by 0.4 mm and are mounted on a shared ground plane, with the entire antenna measuring 0.065 × 0.057 × 0.0042 λ_0_^3^. The designed MIMO antenna functions within the 2.45 GHz ISM band, which has an FBW of 28.16% (2.14–2.83 GHz) and a 3 dB ARBW of 16.3% (2.23–2.63 GHz).

By analyzing the results obtained in a seven-layer homogeneous human head model, a real human head model, and minced pork, this MIMO antenna was determined to be effective. Additionally, the antenna’s low SAR permits operation with an input power of 24.27 mW when considering 10 g of tissue. This setup allows for data transmission rates of up to 78 Mbps across a distance of 9.6 m. Furthermore, the antenna exhibits significant independence between the MIMO units, with excellent performance in terms of ECC, DG, and CC. Therefore, this antenna is well suited for biomedical IMDs that require high data transfer rates.

## Figures and Tables

**Table 1:** Thickness and electromagnetic properties of the 7-layer head model.

Tissue	Thickness (mm)	2450 MHz	
		*ε_r_*	*σ*
Skin	4	38	1.5
Fat	2	5.3	0.1
Muscle	4	52.7	1.7
Skull	5.5	18	0.81
Dura	0.5	42	1.7
CSF	5	66.2	3.5
Brain	30	48.9	1.81

**Table 2:** Communication link budget parameters.

Specification	Variable	Value
Transmitting antenna polarization	Circular	
Receiving antenna polarization	Circular	
Frequency (MHz)	*f*	2450
Implantable antenna gain (dBi)	Gt	−24.1
Transmitted power(dBm)	Pt	−16
Impedance mismatch losses at transmitting side (dB)	Lt	0
Distance (m)	d	0–20
Ambient temperature (K)	T0	293
Rx noise figure	NF	3.5
System noise temperature(K)	Ti	732.5
Rx gain	Gr	2.15
Boltzmann constant	K	1.38 × 10^−23^
Noise power density (dB/Hz)	N0	−199.95
Data rate (Kbps)	Br	Various
Ideal PSK (dB)	Eb/N0	9.6
Coding gain (dB)	Gc	0
Fixing deterioration (dB)	Gd	2.5

**Table 3:** Comparative evaluation of the proposed implantable MIMO antenna with existing implantable MIMO designs.

Parameters	[11]	[16]	[17]	[18]	[35]	[36]	This Work
Size (mm^3^)	13.04	360	3375	9.01	434.6	280	28.448
Frequency (GHz)	0.433	2.45	2.45, 5.8	0.915	2.45	2.4	2.45
Tissue	Skin	Five-layer head model	Skin–fat–muscle	Human body	Skin–fat–muscle	Skin–fat–muscle	Seven-layer head model
Isolation (dB)	26	26.3	37/32	29.7	15.9	37	27.5
ECC	0.1	0.1	0.1	0.1	0.0025	-	0.1
FBW (%)	33.9	0.151	36/26	10.5	18.64	8.5	28.16
Polarization	LP	LP	CP	CP	LP	LP	CP
ARBW (%)	-	-	36/8.33	10.35	-	-	16.3
Polarization diversity	-	-	No	Yes	-	-	Yes

## Data Availability

The original contributions presented in this study are included in the article. Further inquiries can be directed to the corresponding author(s).
